# Genome Sequence of an Endophytic Fungus, *Fusarium solani* JS-169, Which Has Antifungal Activity

**DOI:** 10.1128/genomeA.01071-17

**Published:** 2017-10-19

**Authors:** Jung A Kim, Jongbum Jeon, Sook-Young Park, Ki-Tae Kim, Gobong Choi, Hyun-jung Lee, Yangsun Kim, Hee-sun Yang, Joo-Hong Yeo, Yong-Hwan Lee, Soonok Kim

**Affiliations:** a Genetic Resources Assessment Division, National Institute of Biological Resources, Incheon, South Korea; b Department of Agricultural Biotechnology, Interdisciplinary Program in Agricultural Genomics, Center for Fungal Genetic Resources, and Center for Fungal Pathogenesis, Seoul National University, Seoul, South Korea; c Department of Plant Medicine, Sunchon National University, Suncheon, South Korea

## Abstract

An endophytic fungus, *Fusarium solani* strain JS-169, isolated from a mulberry twig, showed considerable antifungal activity. Here, we report the draft genome sequence of this strain. The assembly comprises 17 scaffolds, with an *N*_50_ value of 4.93 Mb. The assembled genome was 45,813,297 bp in length, with a G+C content of 49.91%.

## GENOME ANNOUNCEMENT

Endophytic fungi are among the most promising natural resources for the discovery of novel bioactive compounds with potential applications in medicine, agricultural chemistry, and the food industry. Additionally, some of these bioactive compounds show anticancer, immunosuppressant, and antimicrobial activities (1, 2). An endophytic fungal strain, JS-169, isolated from a mulberry (*Morus alba*) twig, displayed considerable antimicrobial activity, especially against human fungal pathogens, such as *Candida albicans*, *Candida glabrata*, and *Cryptococcus neoformans*. This strain was identified as *Fusarium solani* based on morphological and molecular analyses. The fungus produced red-to-purple pigments in potato dextrose medium, which is positively correlated with antifungal activity. To aid understanding of the biosynthetic pathways of bioactive compounds, we sequenced the genome of the *F. solani* JS-169.

The mycelia were harvested after overnight incubation in potato dextrose broth at 23°C by static culture. The genomic DNA was extracted using the DNeasy minikit (Qiagen, Valencia, CA, USA). Sequencing was performed using a combination of 1 paired-end and 2 mate pair libraries on a HiSeq 2000 platform (Illumina) and 8 cells on a PacBio RSII platform at Theragen Etex Bio Institute (Suwon, South Korea) covering 460.03-fold of the genome. The reads were assembled using a mixed pipeline of FALCON, an assembler of PacBio reads, and SOAPdenovo version 2, an assembler of Illumina reads (3), which, in turn, were merged using HaploMerger 2 (4). The draft assembly of *F. solani* JS-169 consisted of 17 scaffolds (579 contigs), with an *N*_50_ value of 4.93 Mb. The total length of the assembled genome was 45,813,297 bp, with a G+C content of 49.91%.

The *F. solani* JS-169 genome was estimated to have 15,465 protein-coding genes based on the AUGUSTUS software (5). We identified 1,449 genes encoding secretory proteins, 780 transcription factor genes, 133 cytochrome P450 genes, 9 genes encoding laccases, 86 genes encoding plant cell wall-degrading enzymes, and 27 genes encoding peroxidases using a previously developed gene family pipeline (6–11). A total of 14 polyketide synthases and 26 nonribosomal peptide synthases were also identified through antiSMASH (12, 13). This draft genome sequence will support the identification of genes related to the biosynthesis of antimicrobial compounds and will accelerate research on the biology of endophytic fungi.

### Accession number(s).

The draft genome sequence of *Fusarium solani* JS-169 has been deposited in GenBank under accession no. NGZQ00000000. The version described in this article is the first version, NGZQ01000000.
